# Cost-effectiveness of Family Group Conferencing in child welfare: a controlled study

**DOI:** 10.1186/s12889-018-5770-5

**Published:** 2018-07-09

**Authors:** Sharon Dijkstra, Hanneke E. Creemers, Francisca J. A. van Steensel, Maja Deković, Geert Jan J. M. Stams, Jessica J. Asscher

**Affiliations:** 1 Forensic Child and Youth Care Sciences, University of Amsterdam, Nieuwe Achtergracht 127, 1018 WS Amsterdam, The Netherlands; 2 Developmental and Parenting Problems, University of Amsterdam, Nieuwe Achtergracht 127, 1018 WS Amsterdam, The Netherlands; 3 Child and Adolescent Studies, Utrecht University, Heidelberglaan 1, 3584 CS Utrecht, The Netherlands

**Keywords:** Cost-effectiveness, Family group conferencing, Child welfare, Child safety, Empowerment, Social support

## Abstract

**Background:**

This study aimed to examine the short- and long term (cost-) effectiveness of Family Group Conferencing (FGC) compared to care as usual (CAU) in terms of improved child safety, empowerment and social support.

**Methods:**

A subgroup of a larger randomized controlled trial, comprising 69 families in child welfare (experimental group: *n* = 46; control group: *n* = 23), was included.

**Results:**

No additional effects of FGC on child safety, social support and only short-term positive effects on empowerment were found. There were no differences in costs between FGC and CAU. The chance for FGC to be cost-effective was small. For families who refused FGC, the FGC approach was more cost-effective than CAU, whereas it was less cost-effective for families that prepared or completed FGC.

**Conclusions:**

Overall, FGC is not (cost-)effective in improving child safety, empowerment and social support, but cost-effectiveness varies at different levels of FGC-completion.

**Trial registration:**

Dutch Trial Register number NTR4320. Registered 17 December 2013.

## Background

Worldwide, child welfare agencies are responsible to protect and promote the welfare of children and adolescents. With a yearly budget of 29,1 billion dollars in the United States [1] and 3,5 billion euros in a small country such as the Netherlands [2], child welfare seems to be an expensive endeavor. Fluctuations in available budget play a role in the choices that governments and policymakers make about how children are protected and what services they receive. Without a good impression of the (cost-)effectiveness of interventions and services that are offered in child welfare, it is impossible to make well-informed policy decisions on investments in innovations in care [3]. Therefore, the current study focused on the effectiveness and cost-effectiveness of a method that is increasingly used in child welfare, namely Family Group Conferencing (FGC).

FGC is a decision-making model in which a family together with its extended network (e.g., family members, friends or neighbors) makes its own care plan, that is, a family group plan. The main difference with regular decision-making in child welfare is that the family and the extended network, instead of the child welfare worker and the family, are responsible for both making the plan as well as implementing the plan. This way, FGC aims to establish active responsibility and empowerment of the family and its network [4]. Since it has been argued that FGC contributes to improved child safety [5] and cost savings [6], it may be an attractive model for policymakers to embed in child welfare.

Empirical studies that examined the effectiveness of FGC have demonstrated that FGC does not outperform regular care in improving child safety, as indicated by reductions in child maltreatment, number of out-of-home placements and involvement of child welfare [7, 8]. In terms of the secondary outcome measures, which are targeted in interventions to reduce child maltreatment and out-of-home placement, there is empirical evidence showing that FGC can have a positive effect on empowerment and social support. For instance, Sheets and colleagues [9] found that families and relatives felt more empowered directly after a Family Group Decision Making conference (FGDM; a variation on FGC) compared to families that did not organize an FGDM and Dijkstra and colleagues [10] found that 6 months after FGC, parents felt more empowered than parents who received care as usual (CAU). In the same effect study, the authors found that 1 month after a care plan was made, FGC was more effective than CAU in increasing the number of social support sources. However, this effect was not retained at three and 6 months follow up, and not reflected in higher levels of perceived social support. Interestingly, the level of FGC completion was associated with differential effects in terms of social support and empowerment. Higher program fidelity, which indicates a higher level of FGC completion, was associated with more social support sources but also, marginally, with lower perceived empowerment.

To our knowledge, only one cost-effectiveness study on FGC has been published [11]. In this study, focusing on care for young people with an intellectual disability in the Netherlands, the costs of FGC were weighted against effects in terms of the number of areas of concern in the 12 months following FGC. Results indicated that FGC, as compared to CAU, leads to significantly higher annual care costs, which were mostly explained by the costs of the FGC process (€3829,-). Cost-effectiveness analyses showed that, in the scenario in which there was no extra funding available for a reduction in the number of areas of concern, FGC had a probability of 3% to be cost-effective. An extra investment of 5000 euros for a reduction of one area of concern would lead to a probability of 99% for FGC to be cost-effective. However, since the sample of the Onrust and colleagues study consisted of clients with an intellectual disability, and only families who completed the FGC-process with a conference were included, results cannot be generalized to the population of child welfare.

Given the limited knowledge on cost-effectiveness of FGC in child welfare, it is important to conduct robust cost-effectiveness studies. If FGC is indeed associated with cost savings [6], it may be cost-effective even if it is equally effective as CAU in terms of primary and secondary outcome measures. The aim of the present study was, therefore, to examine effectiveness and cost-effectiveness of FGC at 6 and 12 months after FGC. Based on a previous effect study [10], we selected child maltreatment as indicator of the primary outcome child safety and empowerment and social support as secondary outcomes. Furthermore, previous findings point towards differential effects for different levels of completion of FGC. This suggests that specific elements of the FGC approach are responsible for effects and, possibly, also for cost-effectiveness. Therefore, we also distinguished four subgroups to determine cost-effectiveness for different levels of completion of FGC, namely, 1) families that dropped out in the referral phase of FGC, 2) families that dropped out after an informative meeting, 3) families that dropped out in the preparation of FGC and 4) families that completed FGC with a conference.

In sum, we examined the short- (6 months after FGC) and long term (12 months after FGC) effects of FGC in terms of improved child safety, empowerment and social support and the cost-effectiveness of FGC versus CAU overall, and for different levels of completion of FGC.

## Method

### Participants

This cost-effectiveness study was part of a randomized controlled trial examining the effectiveness of FGC in child welfare in the Netherlands [12]. This FGC-study started with 346 families who were referred to the Child and Youth Care Protection Services (CYPSA) in the period of January 2014 until December 2014. The target group of CYPSA consists of families in which child safety is at stake, mostly families with multi-complex problems in domains such as child maltreatment, mental health, alcohol and other drug problems, high-conflict divorce, delinquency and school problems. The care that is offered to the families is compulsory, and in some families a supervision order has been imposed. For this study, families were randomly assigned to the FGC-group (*n* = 242) or the CAU-group (*n* = 104), in a ratio 2:1. All families referred to the child welfare agency were eligible for participation in this study, as FGC is believed to be suitable for all families. However, of the 346 referred families, 18 families (FGC-group *n* = 5; CAU-group *n* = 5) eventually did not received services of the child welfare agency as, according to the child welfare worker, they did not belong to the target group of the child welfare agency. Those families were removed from the study. Additionally, another 8 participants were removed from the FGC group, as an FGC had never been offered. Data were collected from January 2014 until October 2016 by means of self-report questionnaires and by a risk assessment tool at five measurement occasions: after referral to the child welfare agency (pre-test; T1) and one (T2), three (T3), six (T4) and 12 months (T5) after a care plan had been made. More details about the design of the study, inclusion and procedure are published elsewhere [10, 12].

For the present study, we used data of T1, T4 (short term) and T5 (long term). Only 100 families (30% of the total sample) had complete cost data on T4 and T5. These 100 families did not differ from families without complete cost data (228 families) on ethnic background, divorce status, intellectual disability, referral reason and child maltreatment at T1 (all *p*’s > .05). However, the groups did differ with regard to empowerment at T1; families with complete data felt more empowered than families that had not completed both cost questionnaires. Moreover, within this subsample of 100 families, the FGC-group (*n* = 77) and the CAU-group (*n* = 23) differed significantly on multiple background variables and outcome variables at T1. In order to have comparable groups, *n* = 23 families in the control group were matched to *n =* 46 families in the FGC-group by means of a propensity score [13], based on ethnic background, divorce status, intellectual disability, referral reason and pretest scores on child maltreatment, empowerment and social support.

Thus, the total sample for the current study consisted of 69 families. Propensity score matching was successful as families in the FGC-group did not differ from families in the CAU-group on any of the background characteristics and outcome variables at pretest. Half of the families had a Western background (51%). Around 83% of the biological parents were divorced or lived separately, and 77% of the families were low educated. Less than 10% of the biological parents had an intellectual disability. Around 42% of the families were referred to child welfare due to parental problems (e.g., psychopathology or substance abuse), as opposed to child related problems (26%, e.g., delinquency or school problems) or family related problems (32%, e.g., child maltreatment and neglect) (Table 1).

**Table 1:** Background characteristics of the FGC-group and the CAU-group at pretest (T1)

	Total (*N* = 69)	FGC (*N* = 46)	CAU (*N* = 23)	
	Percent			χ^2^
Ethnicity status				0.03
Western	50.7	50.0	52.2	
Non-western	49.3	50.0	47.8	
Family situation				0.45
Intact families	17.4	15.2	21.7	
Broken and/or newly formed families	82.6	84.8	78.3	
Indication of intellectual disability parent(s)	8.7	10.9	4.3	0.82
Education level of parent(s)				0.65
Lower	76.8	73.9	82.6	
Higher	23.2	26.1	17.4	
Referral reason				1.92
Child related	26.1	23.9	30.4	
Parent related	42.0	47.8	30.4	
Family related	31.9	28.3	39.1	
	M (SD)			t
Mean number of children per family	1.65 (1.08)	1.67 (1.14)	1.61 (0.99)	−0.23
Mean age children involved in child welfare	9.77 (4.72)	10.19 (4.70)	8.93 (4.76)	−1.05
Risk score for child maltreatment	0.18 (0.15)	0.18 (0.15)	0.18 (0.15)	0.08
Number of different types of previous professional care	2.88 (2.96)	2.63 (2.89)	3.39 (3.10)	0.09

Note. *FGC* experimental group - receiving the offer of Family Group conferencing, *CAU* control group - receiving Care As Usual

### Child welfare approach and FGC

After referral to the child welfare agency, the FGC-group as well as the CAU-group were assigned to a child welfare worker and received Intensive Family Case Management (IFCM) [14], a supervision and case management method, based on Functional Family Parole Services (FFPS) [15, 16], for engaging, motivating and working with high-risk youth and multi-problem families. In the first 6 weeks, the child welfare worker established a collaboration with the parents. Then, in the CAU-group, a care plan was made and implemented under responsibility of the child welfare worker. In the CAU-group, it took on average 23 weeks (*SD* = 16.71, range 2–61) to make a care plan in the CAU-group. During the entire research period, families in the CAU-group were not allowed to start FGC.

Families in the FGC-group were offered an ‘Eigen Kracht-conferentie’ [Own Strength conference], the Dutch translation of the original model of FGC. This model consists of four phases. In the referral phase, the child protection worker gives the family the opportunity to organize an FGC together with its extended network. In this phase, an independent coordinator, who is not affiliated with the child welfare service, is matched to the family [17]. In the preparation phase, the FGC-coordinator visits the family for an informative meeting and when the family decides to pursue an FGC, the preparation starts and the coordinator visits the extended network and they all together prepare a conference. The third phase, the conference itself, consists of 1) an information part in which professionals share information on the needs and care options and provide, if necessary, conditions for the plan, 2) a family-deliberation part (private part) in which the care plan is developed (no professionals or coordinator are present) and 3) a presentation part in which the family and the extended network present the care plan to the FGC-coordinator and the professionals. When the care plan is approved by the child protection worker, the family and extended network start the final phase in which the care plan is implemented and implementation is monitored. During the process of FGC, families may dropout before a care plan has been made. In that case, a care plan is made and implemented in the same way a plan is made in the CAU-group, namely under responsibility of the child welfare worker. For families in the FGC-group, it took on average 29 weeks (*SD* = 13.13, range 8–59) to make an FGC-plan or, when families dropped out, a CAU-plan.

### Outcome measures

*Child maltreatment* was scored by the child welfare worker at T1, T4 and T5 and measured on the Actuarial Risk Assessment Instrument Youth Protection (ARIJ) [18]. The ARIJ measures parental risk for child maltreatment in the future and consists of 23 items to be answered on a two-point scale (0 = *absent* and 1 = *present*). For the present study, we selected the items that focused on indications of child maltreatment, namely five items that assess current physical abuse, sexual abuse, emotional abuse, neglect and domestic violence. Answers on these five items were dichotomized (0 = *no indication of child maltreatment*; 1 = *indication of child maltreatment – physical abuse, emotional abuse and/or neglect*). In some families the child welfare worker was no longer involved at T4 (*n =* 20) and T5 (*n* = 34) due to case closure. When these families were not re-referred to the child welfare agency within the research period, indication of child maltreatment was scored 0.

*Empowerment* was assessed by parents at T1, T4 and T5 and measured by the subscale Family of the Family Empowerment Scale (FES) [19]. The subscale Family consists of 12 items rated on a 5-point Likert-type scale (1 = *not true at all* to 5 = *very true*) and assesses parents’ perception of empowerment in parenting situations. A higher mean score means that parents feel more competent about their parenting skills. An example of a question is: “I believe I can solve problems with my child when they happen”. Cronbach’s alpha was 0.78 at T1, 0.81 at T4 and 0.84 at T5.

*Social support* was assessed by parents at T1, T4 and T5 and measured by the short version of the Interpersonal Support Evaluation List (ISEL-short form) [20]. The ISEL-short form consists of 12 items rated on a 4-point Likert-type scale (1 = *definitely false* to 4 = *definitely true*). A lower mean score means that families believe they have limited social support available in their life. An example of a question is: “When I feel lonely, there are several people I could call or talk to”. Cronbach’s alpha was 0.72 at T1, 0.72 at T4 and 0.69 at T5.

### Cost questionnaire and unit costs

To measure the costs associated with child welfare involvement of families in the past 6 months, parents completed the Questionnaire Intensive Care for Youth: health care utilization and productivity loss [21] at T4 and T5. This questionnaire is based on the Trimbos/iMTA questionnaire for costs associated with psychiatric illness [22]. As recommended for economic evaluations [21, 23], it covers the entire social perspective by including costs that are directly related to prevention or treatment as well as indirect costs that result from productivity loss. Our selection for direct and indirect costs was based on previous cost-effectiveness research [24, 25]. In addition, to make this questionnaire more suitable for the target group of child welfare, we added items relating to child welfare involvement (i.e., contacts with social worker, residential/detention care, foster care, contact with police, court or lawyer).

The Questionnaire Intensive Care for Youth consists of three domains. First, direct health care consumption of the children/adolescents in the family is assessed, including contacts with professionals such as psychiatrist, psychologist, social worker, general practitioner, pediatrist, medical specialist, alternative practitioner, hospital care, in-patients care, residential/detention care, day treatment, addiction care, foster care and judicial authorities. Second, direct health care consumption of the parent(s) is assessed, consisting of the same items. Third, indirect non-health care costs are assessed, including absence of work, productivity losses and loss of daily activities for child(ren) and parent(s). Information about the duration of child welfare and use of FGC was derived from case files of the child welfare agency in Amsterdam and from the program bureau organizing FGC in the Netherlands, respectively. Costs of child welfare were calculated from the start of child welfare until case closure. Health care and additional care consumption of families were calculated from care plan until T5.

Costs were calculated by multiplying the number of contacts with the resources used by the unit price of each resource. See Table 2 for an overview of each resource and corresponding unit price. All costs were indexed at 2015. Unit prices for health care were obtained from the Dutch Guidelines for Cost Research [26] and the Manual of the Questionnaire Intensive Care for Youth [21]. Furthermore, unit prices for non-health care were computed based on the overall mean hour productivity costs, derived from the Dutch Guidelines for Cost Research. Unit prices for school absence and loss of daily activities for the child and parent were obtained from the cost-effectiveness study of Van Steensel and colleagues [24]. Unit prices of child welfare were obtained from the child welfare agency in Amsterdam. Additionally, costs of FGC were obtained from the program bureau organizing FGC in the Netherlands. For each family in the experimental group, a cost price of FGC was determined based on the number of completed FGC-phases. Prices were obtained from the Dutch organization “Eigen Kracht Centrale”, that is responsible for the organization of this type of FGC. A price of 0 euro was assigned to families who dropped out in the referral phase, a price of 285 euro was assigned to families who did have an informative meeting but did not start with the preparation phase, a price of 2564 euro was assigned to families who dropped out in the preparation phase, and 3829 euro was assigned to families who completed all phases of FGC including the conference.

**Table 2:** Unit price of each resource

Costs	Resource	Unit price (indexed at 2015 euro’s)
		p/c = per contact
		p/d = per day
Health care costs	Psychiatrist/psychologist	94.90 p/c
	Psychiatrist/psychologist (institutional)	112.67 p/c
	Social worker	71.92 p/c
	General practitioner	33.20 p/c
	Pediatrist	101.61 p/c
	Medical specialist	92.55 p/c
	Alternative practitioner	54.63 p/c
	Emergency aid hospital	260.55 p/c
	In-patient hospital care	505.77 p/d
	In patient mental health care (parent)	256.69 p/d
	Residential/detention care	557.18 p/d
	Day treatment	170.39 p/c
	Addiction care	191.41 p/c
	Foster care	
	Age 0–8	17.65 p/d
	Age 9–11	17.87 p/d
	Age 12–15	19.44 p/d
	Age 16–18	21.49 p/d
	Council for child protection	109.25 p/c
	Police	71.92 p/c
	Lawyer/court	109.25 p/c
Non-health care costs	School absence – regular education	5.33 p/h
	School absence – special education	10.65 p/h
	Loss of daily activities for the child	5.33 p/h
	Loss of daily activities for the parent	12.74 p/h
	Loss of paid work for the parent	34.96 p/h
	Loss of unpaid work for the parent	14.08 p/h
Child welfare	Child welfare	23.79 p/d
Costs of FGC^a^	Completed conference	3829
	Preparation FGC without conference	2564
	Only information meeting	285

^a^ Unit prices are based on the Dutch translation of the original model of FGC, namely ‘Eigen Kracht-conferentie’ [Own Strength conference]

### Analyses

Since the decision of the family to pursue FGC is part of the process of FGC, an intention-to-treat design was applied following the principle of Montori and Guyant [27]. This method was used to eliminate potential confounding effects of treatment motivation. Therefore, all families in the FGC-group were included in the analyses, irrespective of their level of completion of the FGC process. To examine the differences in outcome measures between the FGC-group and the CAU-group, logistic regression analyses were conducted for the outcome child maltreatment and ANCOVA’s were conducted for the outcomes empowerment and social support, accounting for pre-test scores. Mean costs were calculated for both the FGC-group and the CAU-group for the past six (short term) and 12 months (long term) after a care plan was made. Additionally, mean costs were specified for the four subgroups with different levels of completion of FGC. To investigate if cost data were normally distributed, a Kolmogorov-Smirnov test was performed. Due to skewed cost distributions, bootstrap analyses were conducted to determine significant differences in costs between FGC and CAU. Bootstrap analysis randomly draws, with replacement, a number of 1.000 samples of the original data to estimate the sampling distribution of a statistic [28].

In the cost-effectiveness analyses, mean costs in the past 6 months and 12 months were linked to effectiveness in terms of child maltreatment, empowerment and social support. For child maltreatment, effects in the cost-effectiveness analyses were turned into a positive result, namely child safety. For each outcome, three different cost-effectiveness measures were calculated to give an overall view on the cost-effectiveness of FGC, namely an incremental cost-effectiveness ratio (ICER), a cost-effectiveness plane (CE-plane) and a cost-effectiveness acceptability curve (ICEA). By dividing the differences in costs between the two conditions by the differences in effect (Costs FGC – Costs CAU / Effect FGC – Effect CAU), the ICER was computed [23]. Based on this ICER, FGC could be inferior to CAU, which means that FGC shows smaller effects and higher costs, or FGC could be dominant to CAU, which means that FGC shows larger effects and lower costs. In the other two cases (smaller effects and lower costs or larger effects and higher costs), the ICER depicts the costs that have to be invested to gain 1 family without an indication of child maltreatment or to reach 1 point improvement on the scales of empowerment and social support (empowerment scale ranges from 0 (low) to 5 (high) and social support scale ranges from 0 (low) to 4 (high). Subsequently, bootstrapping of the original data with 1000 simulations was used to compute the CE-plane and the ICEA. The CE-plane depicts the 1000 ICERs, divided over four quadrants: a northwest quadrant (NW), which indicates that FGC would be less effective and more costly, a northeast quadrant (NE), which indicates that FGC would be more effective and more costly, a southwest quadrant (SW), which indicates that FGC would be less effective and less costly and, lastly, a southeast quadrant (SE), which indicates that FGC would be more effective and less costly. Additionally, the ICEA is generated to present the probability that FGC would be more effective than CAU over a range of willingness to pay thresholds. Cost-effectiveness analyses were repeated for the various levels of FGC-completion versus CAU.

## Results

### Outcome effects

The effects of FGC on child maltreatment, empowerment and social support are presented in Table 3. Six months after a care plan was made, FGC was equally effective as CAU in reducing child maltreatment (OR = 0.93, 95% CI = 0.26–3.41, *p* = .92). The same result was found 12 months after a care plan was made (OR = 0.85, 95% CI = 0.18–3.99, *p* = .83). Regarding empowerment, families in the FGC-group had a higher level of empowerment at T4 compared to families in the CAU-group, *F*(1, 67) = 4.51, *p* < .05. However, at T5 FGC and CAU were equally effective in terms of empowerment, *F*(1, 67) = 0.22, *p* = .64. Regarding social support, both six and 12 months after a care plan was made no differences between FGC and CAU were found in the level of social support, respectively *F*(1, 67) = 0.70, *p* = .41, and *F*(1, 67) = 1.96, *p* = .17.

**Table 3:** Effects of FGC (*n* = 46) vs. CAU (*n* = 23)

	T1 - pretest				T4 – 6 months after				T5–12 months after			
	n / %	OR	95% CI	d	n / %	OR	95% CI	d^a^	n / %	OR	95% CI	d^a^
Indications of child maltreatment		0.73	0.30–2.31	−0.03		0.92	0.26–3.41	−0.09		0.85	0.18–3.99	−0.13
FGC	18 / 39.1				9 / 19.6				5 / 10.9			
CAU	10 / 43.5				5 / 21.7				3 / 13.0			
	M (SD)	F			M (SD)	F time *group			M (SD)	F time *group		
Empowerment		0.00		−0.02		4.51*		0.52		0.22		0.13
FGC	4.28 (0.50)				4.42 (0.53)				4.24 (0.66)			
CAU	4.29 (0.56)				4.15 (0.57)				4.17 (0.61)			
Social support		0.91		−0.26		0.70		0.31		1.96		0.46
FGC	3.44 (0.43)				3.61 (0.40)				3.58 (0.35)			
CAU	3.55 (0.42)				3.59 (0.36)				3.51 (0.37)			

*Notes.* * *p* < .05. ^a^ Controlled for T1

### Costs

In Table 4 the mean costs for families in the past six and 12 months are presented. Six months after a care plan was made, mean costs for families in the FGC-group (€13.422,08) were not significantly higher than mean costs for families in the CAU-group (€11.813,83), M_incremental costs_ = €1600; CI 97.5% = −€2194 - €7606. Twelve months after a care plan was made, results were comparable; mean total costs (T4 + T5) for families in the FGC-group (€20.192,31) did not significantly differ from mean total costs for families in the control group (€17.925,31), M_incremental costs_ = €2297; CI 97.5% = −€6030 - €13,873.

**Table 4:** Mean costs (€) for families in the FGC-group and families in the CAU-group at T4 and T5

	T4 – 6 months after	T5–12 months after
FGC total	€ 13.422,08	€ 20.192,31
FGC – dropped out in referral phase	€ 11.224,08	€ 14.215,20
FGC – dropped out after informative meeting	€ 10.557,09	€ 16.848,07
FGC – dropped out in preparation phase	€ 22.829,90	€ 28.483,12
FGC – completed with conference	€ 13.090,51	€ 31.024,58
CAU	€ 11.813,83	€ 17.925,31

### Cost-effectiveness

**Fig. 1:**
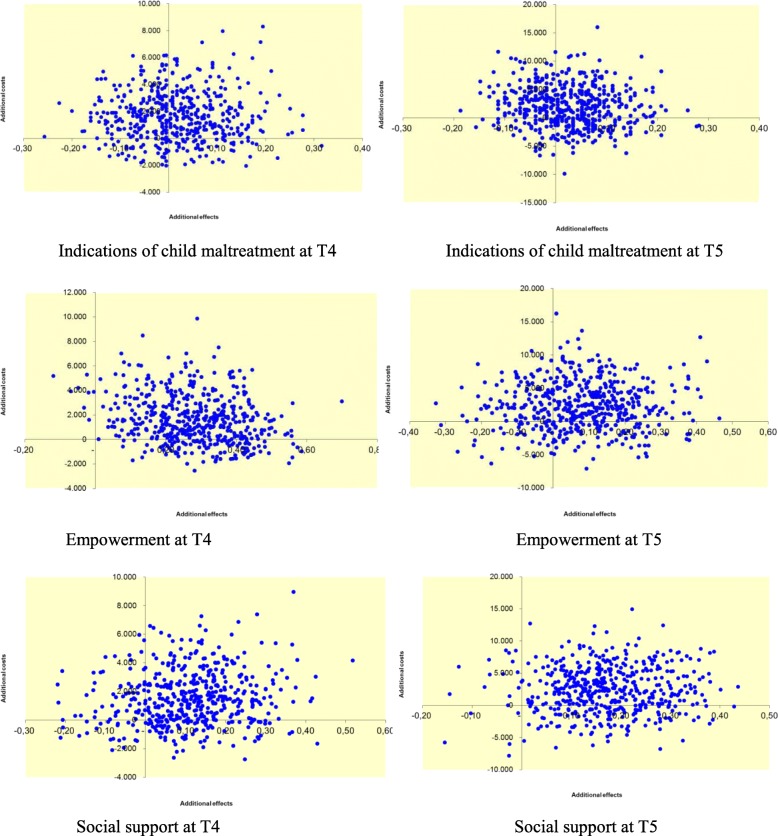
CE-planes of outcomes at T4 and T5

**Fig. 2:**
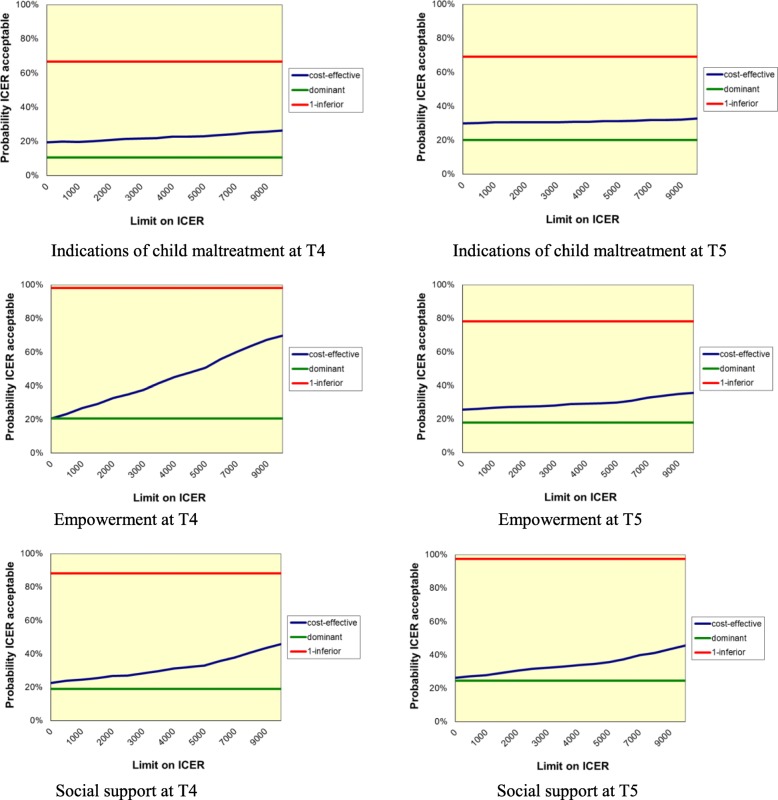
Acceptability curve graphs of outcomes at T4 and T5

Cost-effectiveness results are presented in Table 5 and Figs. 1 and 2. For child safety, the point estimate of the ICER amounted to €80.412,50 at T4 (13.422,08–11.813,83 / 0.80 – − 0.78), which means that with the use of FGC it costed €80.412,50 to gain one family without an indication of child maltreatment. After bootstrapping, the CE-plane showed that the north east quadrant contained most ICERs (47% NE versus 33% NW, 9% SW and 11% SE), indicating larger effects but higher costs. In addition, the ICEA showed that, without additional investments, the chance that FGC would be more cost-effective than CAU was 20% (26% with an investment of 10.000 euro). Similar results were found 12 months after a care plan was made. The use of FGC led to larger effects, but also to higher costs, while the chance that FGC would be more cost-effective than CAU was 30% (33% with an investment of 10.000 euro).

**Table 5:** *Results of the cost-effectiveness analyses – FGC* versus *CAU*

	T4 – 6 months after						T5–12 months after					
	ICER	CE-plane (%)				ICEA curve in %^a^	ICER	CE-plane (%)				ICEA curve in %^a^
Indications of child maltreatment		NE	NW	SW	SE			NE	NW	SW	SE	
FGC total	Inferior	33	49	10	8	18–20	Inferior	25	45	18	12	29–28
FGC – dropped out in referral phase	Dominant	22	4	14	60	74–86	€371.011,-	0	2	58	40	98–95
FGC – dropped out after informative meeting	€41.891,33	4	7	57	32	89–77	€107.724,-	7	26	31	36	67–65
FGC – dropped out in preparation phase	Inferior	19	82	0	0	0–0	Inferior	26	73	1	0	1–1
FGC – completed with conference	Inferior	2	93	5	0	5–0	Inferior	29	71	0	0	1–0
Empowerment												
FGC total	€5.743,75	78	2	0	21	21–70	€28.337,50	53	22	8	18	26–36
FGC – dropped out in referral phase	Dominant	26	0	0	74	74–100	Dominant	2	1	34	64	98–95
FGC – dropped out after informative meeting	Dominant	12	0	0	88	88–100	Dominant	33	1	6	61	66–86
FGC – dropped out in preparation phase	Inferior	51	49	0	0	0–0	€263.945,25	63	36	0	0	1–1
FGC – completed with conference	Inferior	12	81	5	1	6–5	Inferior	46	54	0	0	1–1
Social support												
FGC total	€12.371,15	66	12	4	19	23–46	€13.335,29	71	3	2	25	26–46
FGC – dropped out in referral phase	Dominant	22	3	18	57	75–86	Dominant	2	0	10	88	98–100
FGC – dropped out after informative meeting	Dominant	13	0	1	86	87–99	Dominant	34	0	1	66	66–91
FGC – dropped out in preparation phase	€78.686,21	89	12	0	0	0–0	€42.231,24	99	0	0	1	1–4
FGC – completed with conference	€127.668,-	55	39	2	3	5–21	€261.985,40	75	24	0	0	0–0

*Notes.* ^a^Chance of cost-effectiveness for FGC compared to CAU without additional investments and with investment of 10.000 euro. *ICER* Incremental Cost-Effectiveness Ratio, *CE-plane* Cost-Effectiveness plane, *ICEA* Cost-Effectiveness Acceptability Curve, Dominant: Lower costs and larger effects for FGC compared to CAU; Inferior: Higher costs and smaller effects for FGC compared to CAU

Regarding empowerment, the point estimate of the ICER amounted to €5.743,75 at T4 (13.422,08–11.813,83 / 0.14 – − 0.14), which means that with the use of FGC it costed €5.743,75 to gain one point of improvement in empowerment. After bootstrapping, the CE-plane showed that the north east quadrant contained most ICERs (78% NE versus 2% NW, 0% SW and 21% SE), indicating larger effects but higher costs. In addition, the ICEA showed that, without additional investments, the chance that FGC would be more cost-effective than CAU was 21% (70% with an investment of 10.000 euro). At T5, the use of FGC led also to larger effects, but higher costs, and the chance that FGC would be more cost-effective than CAU was 26% (36% with an investment of 10.000 euro).

With regard to social support, the point estimate of the ICER amounted to €12.371,15 at T4 (13.422,08–11.813,83 / 0.18–0.05). After bootstrapping, the CE-plane showed that the north east quadrant contained most ICERs (66% NE versus 12% NW, 4% SW and 19% SE), indicating larger effects but higher costs. In addition, the ICEA showed that, without additional investments, the chance that FGC would be more cost-effective than CAU was 23% (46% with an investment of 10.000 euro). At T5, the use of FGC led also to larger effects, but higher costs, while the chance that FGC would be more cost-effective than CAU was 26% (46% with an investment of 10.000 euro).

### Cost-effectiveness for different levels of FGC completion

Additional analyses were conducted to specify the cost-effectiveness results for the various levels of completion of FGC (see Table 6 for means and standard deviations among groups). With regard to child safety at T4, we found that for families who dropped out in the referral phase of FGC, FGC was dominant (larger effects and lower costs) to CAU, and had a chance of 74% to be cost-effective compared to CAU. At T5, families in this group showed larger effects but also higher costs, and FGC had a chance of 98% to be cost-effective compared to CAU. Families that had only an informative meeting with an FGC-coordinator showed at T4 larger effects, but also higher costs, with a chance of 89% for FGC to be cost-effective compared to CAU. At T5, FGC was dominant to CAU (larger effects and lower costs), with a chance of 67% for FGC to be cost-effective compared to CAU. However, for families that dropped out in the preparation phase and families that completed a conference, FGC was inferior (smaller effects and higher costs) to CAU with a chance of, respectively, 0 and 5% for FGC to be cost-effective compared to CAU at T4 and a chance of 1 and 1% at T5. Similar results were found for empowerment and social support, which are shown in Table 6.

**Table 6:** Means and standard deviations of FGC divided into four groups

	*n* of subgroups	T1 - pretest	T4 – 6 months after	T5–12 months after
		n / %	n / %	n / %
Indications of child maltreatment				
FGC – dropped out in referral phase	20	5 / 25.0	2 / 10.0	2 / 10.0
FGC – dropped out after informative meeting	10	2 / 20.0	2 / 20.0	1 / 10.0
FGC – dropped out in preparation phase	8	3 / 37.5	2 / 25.0	1 / 12.5
FGC – completed with conference	8	3 / 37.5	3 / 37.5	1 / 12.5
		M (SD)	M (SD)	M (SD)
Empowerment				
FGC – dropped out in referral phase	20	4.34 (0.51)	4.67 (0.41)	4.29 (0.88)
FGC – dropped out after informative meeting	10	3.98 (0.51)	4.29 (0.57)	4.05 (0.56)
FGC – dropped out in preparation phase	8	4.22 (0.40)	4.07 (0.56)	4.14 (0.25)
FGC – completed with conference	8	4.57 (0.44)	4.31 (0.50)	4.44 (0.38)
Social support				
FGC – dropped out in referral phase	20	3.44 (0.49)	3.58 (0.43)	3.55 (0.31)
FGC – dropped out after informative meeting	10	3.38 (0.46)	3.66 (0.31)	3.61 (0.44)
FGC – dropped out in preparation phase	8	3.32 (0.39)	3.51 (0.49)	3.54 (0.42)
FGC – completed with conference	8	3.66 (0.23)	3.72 (0.35)	3.68 (0.29)

### Sensitivity analyses

When searching for outliers in the costs analyses, we found two families in the FGC-group with large costs in the past six and 12 months due to residential care of the child. Although the distribution of children in residential care over the FGC and control group in the subsample was similar to that in the larger study sample, we conducted sensitivity analyses without these two families. Results showed that at T4 costs for the FGC-group reduced from €13.422,08 to €11.816,01, and at T5 from €20.192,31 to €15.985,06. The bootstrapped cost-effectiveness results showed that for child safety at T4, the ICERs were almost equally depicted in two quadrants (16% NW versus 35% NE, 16% SW and 34% SE), with a chance of 50% for FGC to be cost-effective compared to CAU. At T5, most ICERs were found in the south east quadrant (51% SE versus 30% SE, 7% NE and 12% NW), indicating larger effects and also lower costs. The chance for FGC to be cost-effective was 81% compared to CAU. With regard to empowerment, bootstrapped results showed that at T4 the ICERs were almost equally distributed over the south east and north east quadrant (50% SE versus 49% NE, 1% NW and 0% SW), with a chance of 50% for FGC to be cost-effective compared to CAU. At T5, similar results were found. Finally, with regard to social support at T4, the ICERs were almost equally distributed over the north east and southeast quadrant (42% NE versus 38% SE, 10% NW and 10% SW), with a chance of 48% for FGC to be cost-effective compared to CAU. However, at T5 most ICERs were distributed in the south east quadrant (77% SE versus 5% SW, 17% NE and 1% NW), indicating larger effects and lower costs, with a chance of 82% for FGC to be cost-effective compared to CAU.

## Discussion

The current study aimed to provide insight in the effectiveness and cost-effectiveness of FGC in child welfare. First, we examined the short- (6 months after FGC) and long term (12 months after FGC) effects of FGC in terms of improved child safety, empowerment and social support. Second, we examined the cost-effectiveness of FGC versus CAU overall, and for different levels of FGC completion.

With respect to the first aim of the study, we found that six as well as 12 months after a care plan was made, FGC was equally effective as CAU in reducing child maltreatment, which is in line with previous research [7, 8, 10]. Our findings at six and 12 months suggest that effects of FGC in terms of child safety are stable over time. This is in line with the results of a meta-analysis on the effectiveness of FGC, in which no moderator effects were found for duration of follow-up [7]. Furthermore, at the short term, families receiving FGC had higher levels of empowerment than families receiving CAU. However, this effect faded out at the long term. Thus, families seem to benefit from FGC in the short term by feeling more empowered to use their parenting skills, which is in line with previous research of Sheets and colleagues [9] who found that directly after FGC families felt more empowered. However, it seems that this effect is not strong enough to be maintained over a longer period of time. With regard to social support, we found both at short- and long term no differences in effectiveness between FGC and CAU. In an earlier uncontrolled study, FGC was found to be successful in increasing the level of social support [29]. Results from this study indicate that FGC is not superior than CAU in increasing the level of social support. Since previous controlled studies only examined empowerment and social support with a maximum follow-up period of 6 months, no comparisons can be made for long term effects. The results of this study yield, therefore, first insight in the long term effects for FGC on empowerment and social support.

With respect to the second aim of the study, we examined cost-effectiveness of FGC in terms of child safety, empowerment and social support. For child safety, we found no support for cost-effectiveness of FGC; 6 months and 12 months after a care plan was made, FGC led to larger effects but also to higher costs and the chance for FGC to be cost-effective was low, respectively 20 and 30%. With regard to empowerment and social support, we found at both 6 months as well as 12 months after a care plan was made that the chance that FGC would be cost-effective was less than a third. Based on these findings, it seems not cost-effective to implement FGC in child welfare instead of CAU, which is in line with the earlier cost-effectiveness study of Onrust and colleagues [11]. However, whereas Onrust and colleagues found that with an extra investment of 5000 euro, there was a 96% chance that FGC would be cost-effective for reducing the number of areas of concern, we found that even with an extra investment of 10.000 euro, the chance that FGC would be cost-effective increased from 20 to 70%, depending on the outcome measure.

In addition, we examined the cost-effectiveness of FGC by dividing the FGC-group according to the level of FGC-completion; dropout in the referral phase, dropout after an informative meeting, dropout in the preparation phase and no dropout but completion of all phases including a conference. We found that for families dropping out in the referral phase and families dropping out after an informative meeting, FGC had a high chance to be cost-effective compared to CAU for all outcomes on both short and long term (range from 66 to 100%). However, for families who dropped out in the preparation phase and for families who completed all phases including a conference, we found opposite results; smaller effects and higher costs or smaller effects and lower costs. The chance that FGC was cost-effective in these groups was much lower (0–21%).

It seems that families that were offered FGC but refused did benefit more from the FGC approach, compared to CAU, than families who dropped out in the preparation phase or completed all phases. This finding seems in line with the results in an earlier study, in which was found that lower program fidelity (resulting from drop out between the referral and conference phase), was related to higher scores on empowerment [10]. Apparently, when families decided to refuse FGC and to make a care plan with the child welfare worker, this decision resulted in larger effects and sometimes lower costs compared to CAU than when families completed all phases of FGC. Possibly, families who refused FGC have experienced “treatment choice”, an element of the shared decision-making method [30]. A meta-analysis examining the effect of treatment choice in shared decision making on clinical outcomes showed that clients who were involved in shared decision making and chose a treatment condition evidenced higher treatment satisfaction, increased completion rates and superior clinical outcomes, compared to clients who were not involved in shared decision making or did not choose a treatment condition [31]. This would suggest that particularly the offer to use FGC, rather than the FGC approach itself, is (cost)-effective. Further research is needed to examine this hypothesis more specifically.

Because of large costs (due to residential care) in two families in the FGC-group, we performed sensitivity analyses without these two cases. Surprisingly, we found opposite results for cost-effectiveness of FCC at the long term. At short term, FGC still had a low chance to be cost-effective for child safety, empowerment and social support. However, at long term, FGC had a chance of 81% to be cost-effective with regard to child safety and with regard to empowerment and social support, FGC had a chance of respectively 53 and 82% to be cost-effective. It is important to take into account that costs of residential care can have such an influence on cost-effectiveness results. However, placement in residential care is a regular measure that is used in the child welfare system to protect children’s safety and, therefore, part of the health care costs that families make.

### Strength and limitations

In evaluating the significance of the results, it is important to consider strengths and limitations of this study. An important strength of the current study is that, to our knowledge, this is the first prospective cost-effectiveness study on FGC in the field of child welfare. Since FGC is a decision-making model that is implemented in child welfare in numerous countries, it is important to examine the effectiveness of this method carefully. Cost-effectiveness analyses can give more information about the balance between costs and effects. Especially for governments and policy makers, who are responsible for the allocation of the child welfare budget, the current study can be useful. Another strength of the study is the use of multiple measurements. Most cost-effectiveness studies describe the results at one time-point. The present study yields insight in the cost-effectiveness of FGC over time. Finally, we chose to describe cost-effectiveness of FGC for different levels of FGC-completion. Most studies focusing on FGC only include families who completed all phases of FGC and, therefore, not allow for studying the differential effects for various levels of FGC-completion.

This study has also some limitations that should be mentioned. First, we could only use a selection of the larger sample of our randomized controlled trial. This is caused by the fact that we only included families that completed the cost questionnaires at both T4 and T5, which was only 30% of the total sample. Drop-out on this cost-questionnaire was quite large, which may follow from unfamiliarity with this sort of questionnaires in the field of child welfare and the length of the questionnaire. In addition, because of significant differences between the FGC- and the CAU-group in this selection of the sample, we were forced to use propensity score matching to make the groups comparable and, therefore, the sample size further reduced. Although the final sample of *N* = 69 did not differ from the actual sample of *N* = 328 on several background variables (ethnic background, divorce situation, education level, intellectual disability, referral reason), it should be acknowledged that a large number of families was excluded. Second, in the cost-questionnaire only the care costs of one parent were included, mostly the primary care taker. Although the majority of the included families consisted of single parent households and, for these families, the cost-questionnaire covered all costs in the family, 17% of the included families consisted of two parents. For these families, the costs of the other parent, mostly the father, were not covered, which might have resulted in an underestimation of the total costs. Third, in some of the families, the child welfare worker was no longer involved at follow up (*n* = 20 at T4 and *n* = 34 at T5) and, as a result, did not report about child maltreatment. In these families, the presence of child maltreatment was obtained from re-referrals to the child welfare agency and was scored absent when there was no re-referral. Because not all child maltreatment may have been reported, this may have resulted in an underestimation of child maltreatment. Finally, we had no information about the costs that were made by families between the start of child welfare and completion of the care plan. This was caused by the fact that cost-questionnaires were only included at T4 and T5. Although we assumed that differences in costs between the FGC-group and the CAU-group were most likely visible after a care plan was made, it is a limitation that health care costs were lacking for the period between the start of child welfare and the completion of the care plan. Possible effects of FGC and CAU that appeared already before the care plan was made and which could have had an effect on the health care consumption of families, were not taken into account in this study.

### Implications

Despite these limitations, the present study yields important implications for future research and practice. For future research, it is important that more cost-effectiveness studies on FGC be conducted to confirm or disconfirm the results of the current study. Cost-effectiveness researchers have to be aware of high dropout rates in child welfare when adding cost questionnaires to their measurements, and may think of ways to support parents in completing cost questionnaires. In addition, future research into the process of FGC can be relevant. For both effectiveness and cost-effectiveness, we found indications that there are differences in effectiveness between families who immediately refuse an FGC and families who completed all phases of FGC. Since in most previous research only families were examined that completed all phases of FGC, families that did not complete FGC with a conference are underrepresented. Especially because we found that these families benefitted more than families who completed all levels of FGC, is it relevant for future research to focus on these families.

Implications for practice mostly focus on the implementation of FGC. At the moment, FGC is used in child welfare as a replaceable decision-making model of CAU. Based on the results of this study, FGC is not a cost-effective alternative to CAU. Even with extra investments, FGC has a small chance to be cost-effective compared to CAU. The question is how much policy makers want to invest to benefit from FGC, given our findings that FGC and CAU are equally effective in improving child safety, empowerment and social support at the long term. Based on this study, high investments are needed to gain meaningful improvements in child safety, empowerment and social support with the use of FGC. When improved effectiveness is not pursued, FGC could be made more competitive to CAU in terms of cost-effectiveness by reducing the costs directly relating to the FGC. In the current study, the costs of using this particular type of FGC are 4000 euros per family. Reducing these costs would result in a better cost-effectiveness balance. For example, the previous cost-effectiveness study of Onrust and colleagues [11] showed that when controlling for the costs of FGC itself, significant differences in costs between FGC and CAU disappeared. In addition, the longer period of time needed to establish a care plan in FGC may delay improvement in the family situation and extend the period of involvement of child welfare services, which increases child welfare costs. Although it is one of the principles of FGC to give responsibility back to the family and its social network, also with regard to duration of commencing a plan, more boundaries on time to make an FGC-plan may be helpful to reduce costs.

## Conclusion

In conclusion, the findings of this study indicated that, both at short and long term, no effects for FGC were found in terms of child safety and social support and the chance that FGC was cost-effective compared to CAU was low. For empowerment, FGC was more effective than CAU at short term but not at long term, and its chance to be cost-effective was also low. With extra investments, the cost-effectiveness of FGC did somewhat increase. When examining cost-effectiveness for different levels of completion of FGC, we found that for families who refused FGC immediately or after an informative meeting, the FGC approach was more cost-effective than CAU, whereas it was less cost-effective for families that prepared or completed a conference. Since the current study is one of the first cost-effectiveness studies on FGC, it gave first insights in the cost-effectiveness of FGC and the differences in cost-effectiveness between different levels of FGC completion.

## Abbreviations

ARIJ: Actuarial Risk Assessment Instrument Youth Protection
CAU: Care As Usual
CE-plane: Cost-Effectiveness plane
CYPSA: Child and Youth Care Protection Services
FES: Family Empowerment Scale
FFPS: Functional Family Parole Services
FGC: Family Group Conferencing
FGDM: Family Group Decision Making
ICEA: Cost-Effectiveness Acceptability Curve
ICER: Incremental Cost-Effectiveness Ratio
IFCM: Intensive Family Case Management
ISEL: Interpersonal Support Evaluation List
NE: Northeast quadrant
NW: Northwest quadrant
SE: Southeast quadrant
SW: Southwest quadrant
